# Supplementary material for: Conservation Genetics of the Critically Endangered Southern River Terrapin (Batagur affinis) in Malaysia: Genetic Diversity and Novel Subspecies Distribution Ranges
Source: Biology (Basel). 2023 Mar 29;12(4):520. doi: 10.3390/biology12040520 (PMC10136168; doi:10.3390/biology12040520)

Supplementary materials

Fig. S1 (a) Maximum Likelihood (ML) tree showing the relationship among D-loop haplotypes of Batagur affinis, Batagur borneoensis and Dermochelys coriacea. The number at each node represents the bootstrap value % based on 1000 pseudoreplication for the ML analysis. **(b)** Maximum Parsimony (MP) Tree showing the relationship between the D-loop haplotypes of Batagur affinis, Batagur borneoensis and Dermochelys coriacea. The number at each node represents the bootstrap value % based on 1000 pseudoreplication for the MP analysis.

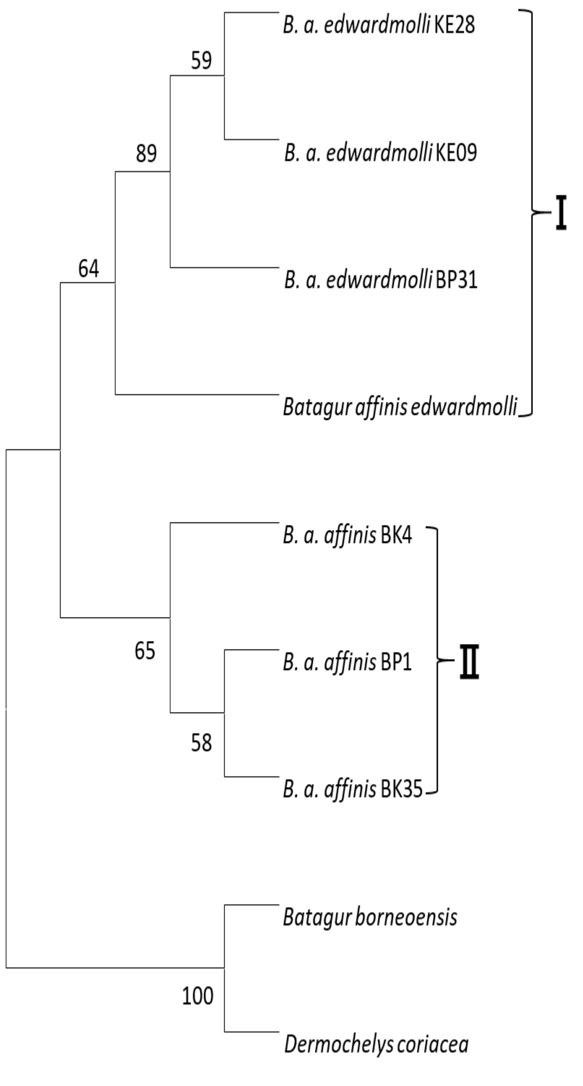

(a)

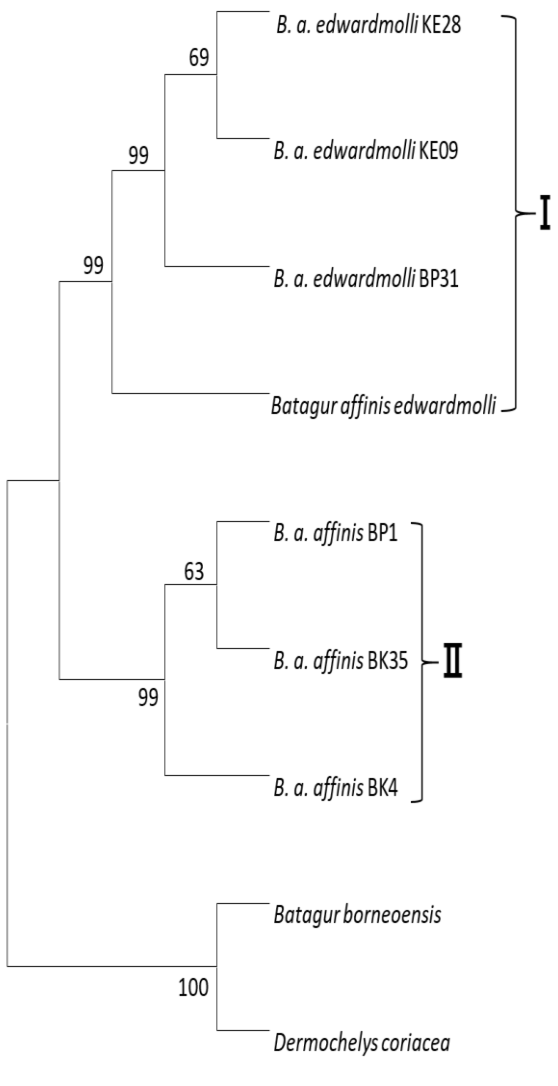

(b)

Fig. S2 The plot of transition (X) and transversion (Δ) against divergence using the F84 distance method.

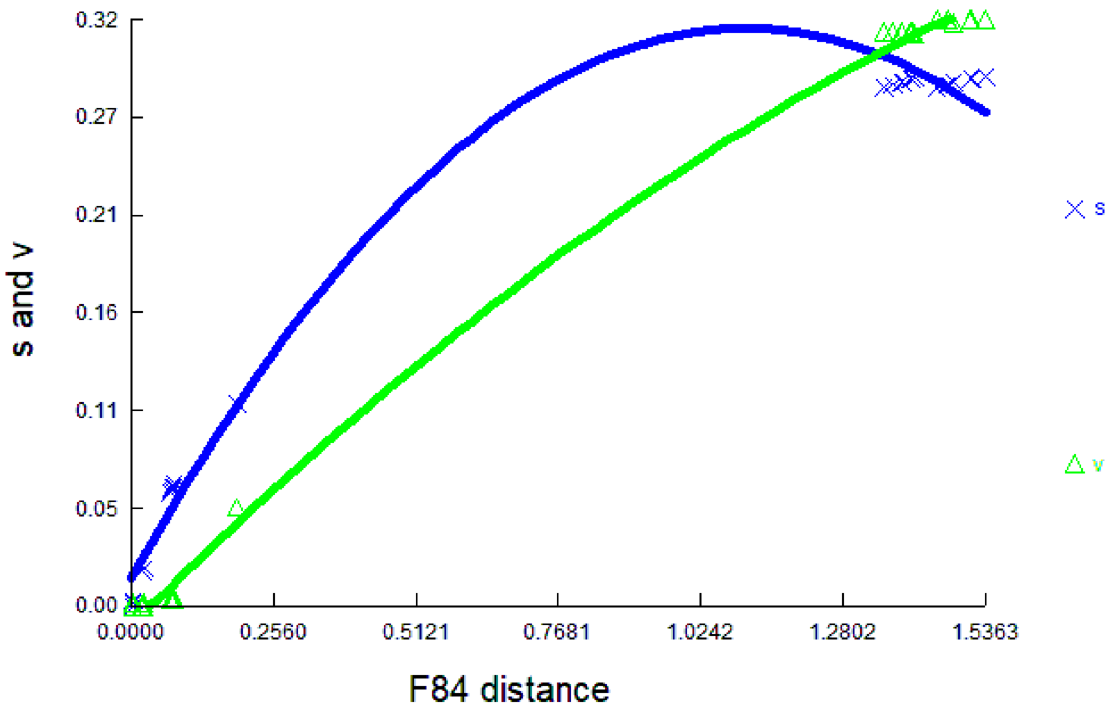

Supplement: Supplementary file 1 [file biology-12-00520-s001.zip › biology-2194897-supplementary.pdf]
